# Experimental Analysis of Smart Drilling for the Furniture Industry in the Era of Industry 4.0

**DOI:** 10.3390/ma17092033

**Published:** 2024-04-26

**Authors:** Krzysztof Szwajka, Joanna Zielińska-Szwajka, Tomasz Trzepieciński

**Affiliations:** 1 Department of Integrated Design and Tribology Systems, Faculty of Mechanics and Technology, Rzeszow University of Technology, ul. Kwiatkowskiego 4, 37-450 Stalowa Wola, Poland; 2 Department of Component Manufacturing and Production Organization, Faculty of Mechanics and Technology, Rzeszow University of Technology, ul. Kwiatkowskiego 4, 37-450 Stalowa Wola, Poland; j.zielinska@prz.edu.pl; 3 Department of Manufacturing Processes and Production Engineering, Rzeszow University of Technology, al. Powstańców Warszawy 8, 35-959 Rzeszów, Poland; tomtrz@prz.edu.pl

**Keywords:** cutting resistance, drilling, material identification, permutation entropy, smart drilling

## Abstract

The fact is that hundreds of holes are drilled in the assembly process of furniture sets, so intelligent drilling is a key element in maximizing efficiency. Increasing the feed rate or the cutting speed in materials characterized by a higher machinability index is necessary. Smart drilling, that is, the real-time adjustment of the cutting parameters, requires the evolution of cutting process variables. In addition, it is necessary to control and adjust the processing parameters in real time. Machinability is one of the most important technological properties in the machining process, enabling the determination of the material’s susceptibility to machining. One of the machinability indicators is the unit cutting resistance. This article proposes a method of material identification using the short-time Fourier transform in order to automatically adjust cutting parameters during drilling based on force signals, cutting torque and acceleration signals. In the tests, four types of wood-based materials were used as the processed material: medium-density fiberboard, chipboard, plywood board and high-pressure laminate. Holes with a diameter of 10 mm were drilled in the test materials, with variable feed rate, cutting speed and thickness of cutting layer. An innovative method for determining the value of unit cutting resistance was proposed. The results obtained were used to determine the machinability index. Based on the test results, it was shown that both the selected signal measures in the time and frequency domains and the unit cutting resistance are constant for a given material of a workpiece and do not depend on the drilling process parameters. In this article, the methodology is proposed, which can be used as an intelligent technique to support the drilling process to detect the material being machined using data from sensors installed on the machine tool. The work proposes the fundamentals for material identification based on the analysis of force signals and the magnitude of force derivatives. The proposed methodology shows effectiveness, which proves that it can be used in intelligent drilling processes. Hybrid wood-based material structures consisting of different materials are becoming more and more common in building structures for strength, economic and environmental reasons. Due to the difference in the machinability of interconnected materials, cutting parameters must be optimized in real time during machining. Currently, with the rapid development of Industry 4.0, the on-line identification of parameters is becoming necessary to improve the process flow in industrial reality. The proposed methodology can be used as an intelligent technique to support the drilling process in order to detect the material being processed using data from sensors installed on the machine tool.

## 1. Introduction

Wood-based panels are becoming more and more widely used in many areas of industrial production [1,2,3,4]. Their growing popularity is determined by numerous advantages over natural wood [5]. Wood-based materials are much cheaper, more homogeneous and isotropic, more resistant to fungi, insects, etc. [4,6,7]. Moreover, they allow the creation of flat surfaces of any dimensions—which is impossible in the case of solid wood. Therefore, the fact that the capabilities of modern wood-based panels can be adapted to specific applications, along with their strength properties and affordable price, make them a real solution for reducing the demand for solid wood [5,8,9,10,11].

The use of wood-like panels in the furniture industry or other industrial fields involves machining them, primarily drilling [12,13]. Therefore, currently, testing the machinability of wood-based panels when drilled seems to be a significant issue [14,15]. Only on the basis of (strictly defined and experimentally determined) relative machinability indices can the machinability of different materials be compared—not only qualitatively, but also quantitatively. This type of comparison, made for different types of wood-based panels, seems to be very important from the point of view of designers, producers and potential users, especially when an innovative wood-based material is to be developed [16,17,18]. Unlike metalworking, there are no standardized machinability tests for wood-based panels. There are relatively few studies comparing the machinability of different materials with different properties. Manufacturers of wood-based materials usually only provide their mechanical properties. As a rule, there is no clear (quantified) information on the machinability of these materials, while the machinability aspect of any structural or decorative material is an important factor that can influence various phases of production, including product design and the planning of production processes.

The hybrid structure of wood-based materials has become essential in building structures for economic and environmental reasons. Due to the different materials combined in composite structures, the cutting parameters must be changed during machining to enable machining optimization [19,20,21]. In the past, the procedure of optimizing cutting parameters was omitted in the production process. Currently, with the rapid development of the Industry 4.0 concept, the identification of parameters in real- time conditions is becoming necessary to improve the machining process in industrial practice [22,23].

Smart factories are a key feature of the Industry 4.0 concept. The Industry 4.0 approach focuses on creating intelligent products, processes and procedures [24] with an emphasis on sustainable development [25,26]. The essence of the process is comprehensive factory management, which allows for the reduction of erroneous factors. In such an environment, there is more efficient production and communication between people, machines and resources, in accordance with the principles of a social network [27,28]. In Industry 4.0, it is assumed that standard jobs will be replaced by artificial intelligence and robots [21,29]. The vision of the future of the European industry resulted in the inauguration of the Industry 5.0 Community of Practice (CoP 5.0) on 16 November 2023, which places even greater emphasis on putting the wellbeing of the worker at the center of the production process [30].

The enormous and continuous increase in the possibilities of using computers since the early 1990s and the simultaneous decline in their prices have resulted in more and more manufacturers using IT techniques to control all phases of the production process. There are many ways to process wood-based panels [7,31,32], so the machinability of these materials can be tested in many ways. Nevertheless, the machinability of any type of wood-based panel when drilled is one of the most important issues from a practical point of view. This general belief can be proven in many ways, but it seems that two basic arguments suffice. Firstly, resistance to axial screw removal is one of the most important technical parameters characterizing wood-based panels. The experimental procedure requires drilling an appropriate hole in order to install the screw, which is why drilling is a very basic form of chipboard processing. Secondly, drilling is now not only used to make holes in the furniture industry. Drilling tests are widely considered the most convenient (fastest and most economical) method for the relative assessment of the machinability of any wood or wood-based materials [33,34]. Any scientific study of machinability must be strictly experimental. In general, it has long been known that any attempts to theoretically determine machinability based on the mechanical properties of the material are imprecise [31,35]. This may contradict the belief that, for example, knowledge of the tool geometry, cutting parameters and standard material properties allow for the theoretical determination of the cutting forces. Previous investigations show that real machining processes, such as drilling, are too complex from a physical point of view to find a direct relationship between the cutting forces and the tensile or shear strength of the processed material [31,32,35]. We are simply forced to conduct experimental research. Unfortunately, there is no generally accepted standard that can be directly applied to testing the machinability of wood-based materials. One of the most reliable test procedures (which can be used for drilling wood-based panels) was proposed and tested by Podziewski et al. [31]. The procedure takes into account two basic aspects (criteria) of machinability: hole quality and cutting force. This is due to the fact that Podziewski et al. [31] (after consulting scientists dealing with cutting theory and woodworking engineers) concluded that these are the only two basic criteria that are important when drilling in wood-based materials. The problem of machining quality may significantly limit the scope of application of the construction material, and excessive drilling resistance may result in the need to limit the feed rate and reduce machining efficiency.

This article proposes methods for identifying the material being processed in the process of drilling four types of wood-based materials using the short-time Fourier transform (STFT) analysis of the acceleration signals measured in the X, Y and Z directions. A simplified method for determining the value of the unit cutting resistance is also proposed, which can be used in the process of identifying the cut material. Additionally, using the permutation entropy measure of the time series of thrust force, its effectiveness and usefulness in identifying the machined material is demonstrated. The aim of the research was to propose the basis of an approach to the automatic (on-line) adjustment of cutting parameters during machining based on selected signal measures in the time and frequency domains.

## 2. Experimental Procedure

### 2.1. Test Materials

**Figure 1:**
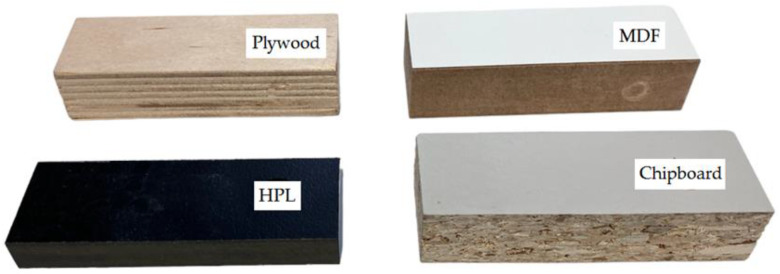
The test materials.

In the tests, four types of wood-based materials were used as the test materials: medium-density fiberboard (MDF), chipboard, plywood board and high-pressure laminate (HPL). Figure 1 shows the materials processed during the tests. The first three materials are the most frequently used materials in the furniture industry. HPLs, on the other hand, are composed of pressurized wood fiber laminated layers, which, thanks to high density and hardness, are very resistant to damage. HPLs are characterized by a durable coating and resistance to fading and mechanical damage. This, in turn, makes it suitable for a wide range of applications in the furniture industry. Before starting the basic research, measurements of the selected mechanical and physical properties of the processed materials were carried out. The density of the HPL was measured in accordance with the ISO 1183-1 [36] standard, and, for the other materials, the EN 622-5 [37] standard was used. Mechanical properties for HPL were determined in accordance with the EN ISO 178 [38] standard, and, for the other materials, the EN 310 [39] standard was used.

**Figure 2:**
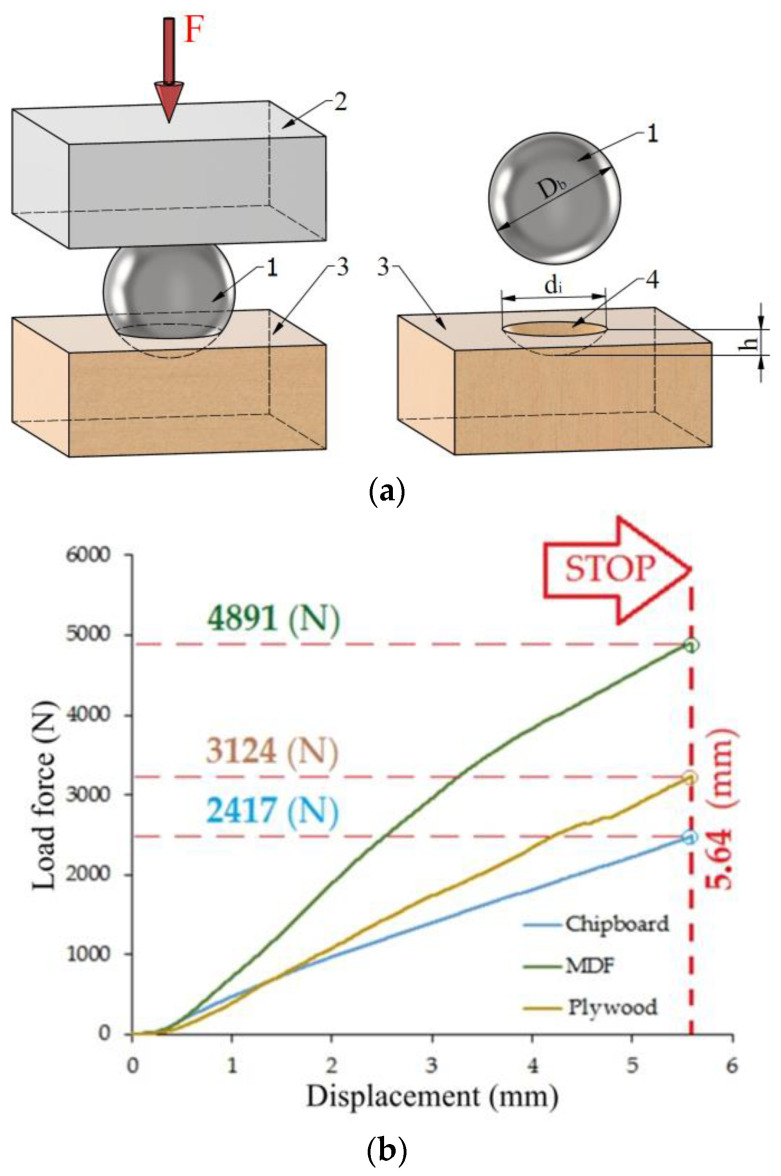
Results of the Janka ball test: (**a**) research methodology, (**b**) research results.

The Janka hardness value (Figure 2) was determined using a Janka ball test with a steel ball with a diameter of 11.28 mm, according to ASTM D 1037 [40] standard. The load was applied continuously throughout the test, with a uniform speed of movement of the moving crossbar of the universal testing machine of 5 mm/min. As a measure of hardness, the maximum load required to push the ball into the wood to a depth of half the ball’s diameter was recorded. The materials used in the research are characterized by a clear variation in material density in the cross-section [41], which results from their characteristic multi-layer structure.

**Figure 3:**
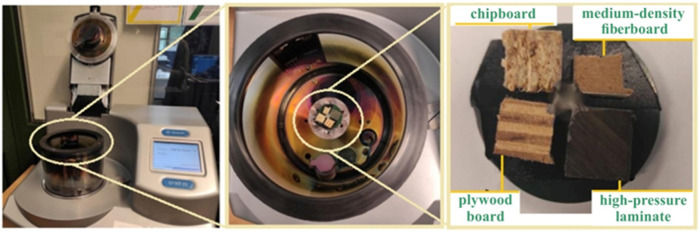
High-vacuum sputtering machine Q150.

In order to visualize the structure and analyze the chemical composition, the test materials were analyzed using a TESCAN^®^ scanning electron microscope (TESCAN, MIRA3, Brno, Czech Republic). Before starting measurements using a scanning electron microscope (SEM), it was necessary to properly prepare the samples. Due to the fact that the materials are dielectrics, it was necessary to apply (spray) a thin layer of electrically conductive material on them. Samples of test materials were placed in a Memmert forced-air drying oven (Memmert GmbH, Buchenbach, Germany) for 15 min at a temperature of 60 °C to evaporate the acetone used to clean the samples and prepare the samples for the sputtering process. Then, the acetone-dried samples were placed in a Q150 (Quorum, San Jose, CA, USA) high-vacuum sputtering machine in order to apply a thin layer of copper to the surface of the samples, which helps to improve electrical conductivity (Figure 3) during SEM examination. The samples were sputtered with copper for 60 s, the current intensity during sputtering was 60 mA. The sputtered samples were placed in a SEM equipped with an EDS (energy dispersive X-ray spectrometer) from TESCAN^®^ (MIRA3, Brno, Czech Republic), and qualitative and quantitative measurement of the chemical elements were carried out. The results of the tests are presented in Table 1. As can be seen, the materials used in the tests are characterized by quite significant differences in mechanical properties. The mechanical properties of materials are fundamental to understanding the behavior of materials in machining processes. 

**Figure 4:**
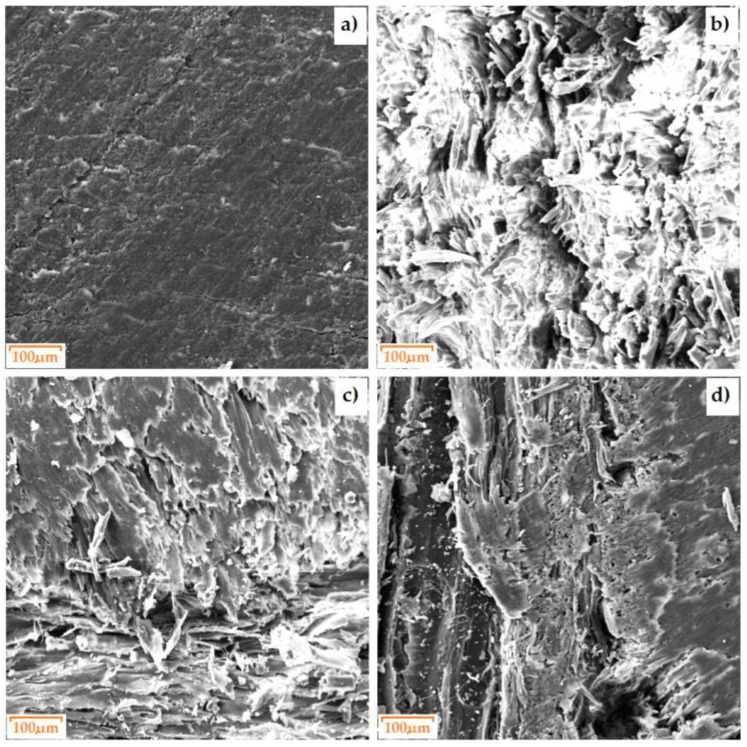
SEM micrographs of the surfaces of the test materials: (**a**) HPL, (**b**) MDF, (**c**) chipboard and (**d**) plywood board.

**Figure 5:**
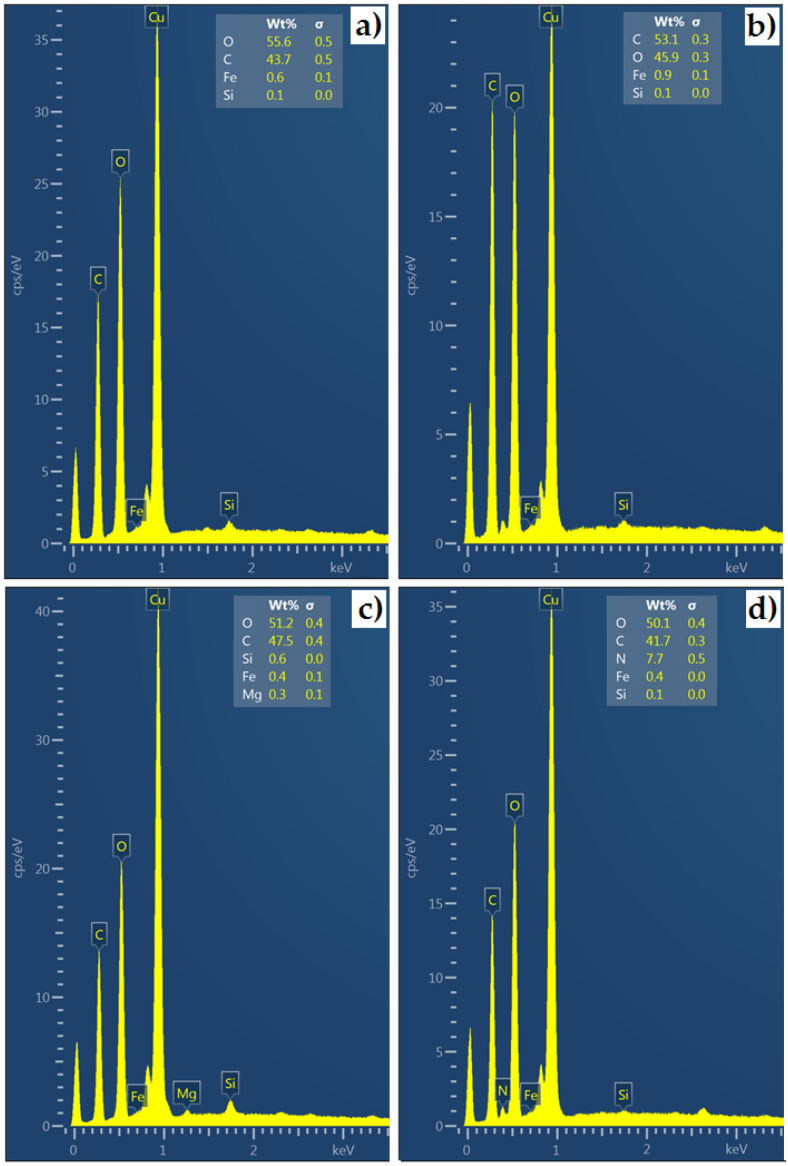
An EDS spectrum of (**a**) chipboard, (**b**) MDF, (**c**) HPL and (**d**) plywood board.

The structures of the processed materials differ from each other. Figure 4 shows the different arrangements of the structures and the differing densities, which translate into, among other things, the hardness of the material. The HPL is characterized by a uniform arrangement of layers. For the plywood board, layers of varying density can be noticed. MDF has a medium-density structure. The internal structure of chipboard is irregular. After the EDS analysis (Figure 5), it can be seen that the following elements are present in all the materials: oxygen, carbon, iron and sulfur. For HPL, the percentage of carbon is the highest compared to other materials and is approximately 47.5%. The presence of magnesium was noticed in the composition of the HPL material.

### 2.2. Cutting Tool

**Figure 6:**
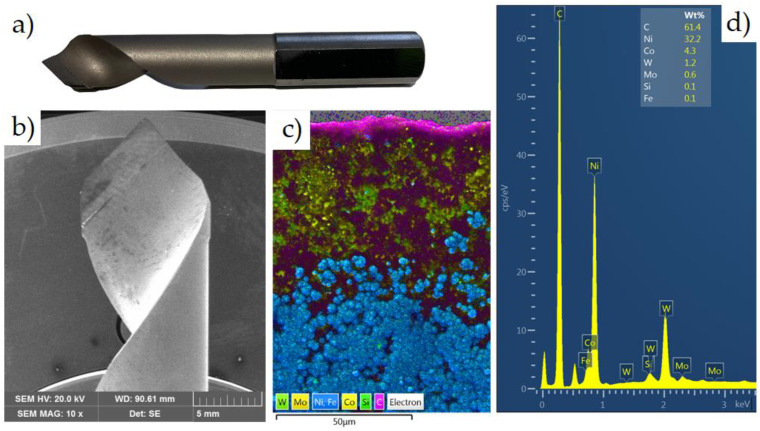
(**a**) View of the cutting tool, (**b**) drill at ×10 magnification, (**c**) element mapping analysis and (**d**) EDS spectrum.

The tests used a typical cutting tool intended for wood-based materials, a single-edge drill with a diameter of 10 mm with polycrystalline diamond (PCD) blades from Leitz^®^ Diamaster PRO (Leitz, Oberkochen, Germany). The drill marking is DP/D10/NL30/S10x27/GL70/RL. This drill is used in processing materials such as wood, chipboards, resin boards, plastics and reinforced plastics. As in the case of the machined material, a spectral analysis of the elements constituting the cutting tool material (Figure 6a,b) was performed using a TESCAN^®^ SEM (TESCAN, MIRA3, Brno, Czech Republic).

After the EDS analysis (Figure 6d), it can be seen that the dominant elements are carbon, nickel, cobalt and tungsten. PCD is a material produced by polymerizing diamond micropowder with metallic binders (such as Co, Ni, etc.). During the sintering process, thanks to the addition of additives, a connecting bridge is created between PCD crystals, the main components of which are Co, Mo, W, WC and Ni (Figure 6c), and the diamond is permanently embedded in the strong skeleton resulting from bonding.

### 2.3. Equipment and Machining Conditions

**Figure 7:**
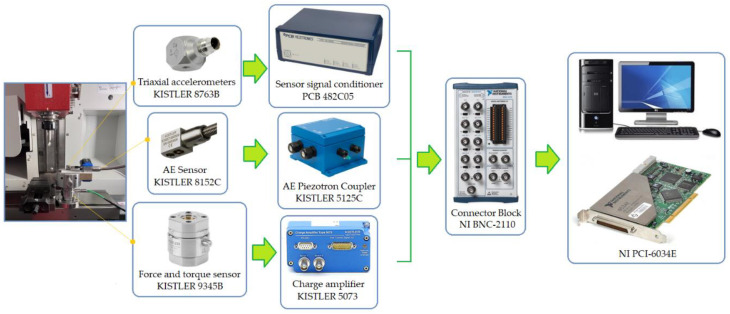
Experimental set-up and schematic of the data acquisition system.

The drilling process was carried out on an EMCO^®^ CNC vertical milling machine (EMCO GmbH, Hallein, Austria). A schematic diagram of the configuration of the measurement track and the measurement data archiving system is shown in Figure 7. During the drilling process, acceleration signals in the directions a_x_, a_y_ and a_z_, thrust force F_t_ and cutting torque M_s_, and an additional acoustic emission signal AE_RMS_ were recorded with an integrating constant τ = 0.12 ms.

As part of the research, holes were drilled on a CNC milling machine in prepared samples (Figure 1), cut from plywood board, MDF and chipboard, with dimensions of 130 × 30 × 18 mm. The thickness of the HPL was 10 mm. The acceleration signal value in three mutually perpendicular directions was measured using a KISTLER^®^ 8763B piezoelectric acceleration sensor (KISTLER, Winterthur, Switzerland), which was mounted on the workpiece (Figure 7). The thrust force F_t_ and cutting torque M_s_ were measured using a KISTLER^®^ 9345B sensor (KISTLER, Winterthur, Switzerland). The acoustic emission signal was measured using a KISTLER^®^ 8152C sensor (KISTLER, Winterthur, Switzerland). Signals from the sensors were recorded on the hard drive of a personal computer in digital form via a National Instruments^®^ 6034E (Austin, TX, USA) analogue-to-digital card. The sampling frequency of signals during the experiments was 50 kHz, and the measurement resolution of the card was 16 bits.

**Figure 8:**
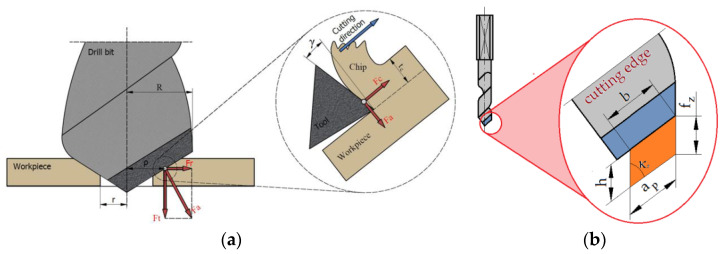
(**a**) Distribution of the cutting forces during redrilling and (**b**) parameters of the cutting layer.

Table 2 and Table 3 summarize the cutting parameters used during the drilling experiments. Three repetitions were performed for each set of cutting parameters. The studies were divided into two groups. In the first group, holes were drilled in the solid material with the parameters shown in Table 2, recording all signals (Figure 7) measured during the cutting process. However, in the second group (Table 3), first, a hole with a diameter of 3 mm was drilled and then, redrilling was carried out with a drill with a diameter of 10 mm. In this case, only the thrust force and cutting torque signals were recorded. The above-described research methodology was repeated for all four types of materials machined with both groups of adopted cutting parameters. This allowed for the experimental determination of the cutting resistance. Figure 8 shows the distribution of cutting forces during redrilling.

After a preliminary analysis, it was determined that, in the case of redrilling in wood-based materials, an effective solution would be to carry out the redrilling process using different thicknesses of the cutting layer. It was assumed that, to determine the cutting resistance for each of the test materials, it would be optimal to determine seven values of the thickness of the cutting layer, starting at 0.05 mm and increasing successively in 0.05 mm steps up to 0.35 mm. For each cutting depth, the feed per tooth and the feed rate increased similarly (Table 3).

Before starting the research, the basic geometry of the drill cutting edge was determined. The drill lip clearance was κ_r_ = 45°, which means that sinκ_r_ = 0.707. Then, the width of the cutting layer (b) was determined, which was a constant parameter for all the drilling tests: b = a_p_/sinκ_r_ = 4.95 mm. To carry out the tests, it was necessary to calculate the feed per tooth f_z_ and feed rate v_f_ for each cutting layer thickness (Table 3). 

## 3. Results

### 3.1. Cutting Resistance

Machinability is a concept describing a set of indicators and criteria determining the machinability of a material under specific conditions, while the ability of a tool to perform machining is defined by the cutting ability. Both parameters depend primarily on the type of cutting process, the blade geometry, and the material of the workpiece. Machinability and cutting ability indicators are selected to suit specific needs. So far, no clear quantitative indicator of machinability has been developed. The basic and most frequently used indicators include the cutting speed and the surface roughness parameters Ra and Rz. A material that is well machinable is one that can be processed at a high periodic cutting speed while maintaining low surface roughness during finishing. These criteria are often contradictory. Therefore, durability and smoothness indicators are usually treated separately. The method of determining machinability using the cutting resistance is used especially in drilling, in particular when drilling small holes. In this case, care should be taken to reduce the cutting resistance, for example, by selecting the appropriate blade geometry. The main reason for this is the tight balance between the cutting torque and the strength of the tool, which can lead to tool damage.

The forces acting in the shear zone do not depend directly on the tool type, but only on the workpiece material, so it is worth trying to determine this relationship. Based on measurements of the force parallel to the shear plane F_s_ and the cutting force F_c_ per millimeter of the width of the cutting layer as a function of the length of shear zone l_s_, Das and Tobias [41] noticed that these lie along a straight line for a wide range of cutting parameters, blade geometry, blade material, and other cutting conditions. It was found that the forces F_s_ and F_c_ depend only on the material being processed. Equations of straight lines can be presented in the following form [34]:

(1) $$F_{s}=F_{sw}+F_{sk}=k_{sw}A_{s}+K_{sk}b$$

(2) $$F_{c}=F_{cw}+F_{ck}=k_{cw}A_{s}+K_{ck}b$$

where A_s_ = b × l_s_ is the area of the shear plane; K_sk_ is the unit component of the cutting force (per 1 mm of the cutting edge length), projected onto the direction of the shear plane; k_sw_ is the unit shear force (per 1 mm^2^ of shear surface area), representing specific shear resistance; K_ck_ is the unit component of the cutting force (per 1 mm of cutting edge length), projected onto the direction of cutting speed; and k_cw_ is the unit cutting force (per 1 mm^2^ of shearing surface area), representing main shear resistance. 

As could be expected, the cutting forces are proportional to the length of the cutting edge (equal to the width of cutting layer), and the forces resulting from the process of turning the cutting layer into a chip are proportional to the area of the shear plane. The specific shear resistance k_sw_ can, therefore, be considered, to some approximation, as a material constant [33].

The main shear resistance k_cw_ is, like the specific shear resistance k_sw_, approximately, a material constant. The ratio k_cw_/k_sw_ is therefore also constant for a given workpiece material, and it has been called the universal machinability index D [34]:

(3) $$D=\frac{k_{cw}}{k_{sw}}=const$$

The area of the shear plane depends on the cross-sectional area of the cutting layer and the shear angle ∅ [34]:

(4) $$A_{s}=bl_{s}=b\frac{h}{sin\emptyset }$$

Substituting (4) into (2), we obtain the physical formula for the cutting force:

(5) $$F_{c}=F_{cw}+F_{ck}=bh\frac{k_{cw}}{sin\emptyset }+bK_{cw}$$

The orthogonal force F_o_, according to the Das and Tobias model, is described by the formula [41]

(6) $$F_{o}=\frac{Dcos\emptyset -1}{Dsin\emptyset }$$

The specific cutting resistance k_c_ is defined as the ratio of the main component of the cutting force F_c_ to the cross-sectional area of the cutting layer A_D_:

(7) $$k_{c}=\frac{F_{c}}{A_{D}}$$

To understand the dependence of the specific cutting resistance k_c_ on the thickness of the cutting layer, the specific cutting resistance can be represented by two components:

(8) $$k_{c}=\frac{K_{ck}}{h}+\frac{k_{cw}}{sin\emptyset }=k_{c1}+k_{c2}$$

The first of these, being the ratio of the force K_ck_ per millimeter of the active length of the cutting edge to the thickness of the cutting layer h, plays an important role only for small thicknesses of the cutting layer. For larger thicknesses of the cutting layer, this component is insignificant, and the main role is played by the second component, which depends on the shear angle ∅. As it is known, the shear angle increases with the thickness of the cutting layer, which results in a slight reduction in shear resistance. The specific cutting resistance is not determined by Equation (8), but experimentally, directly from Equation (7), along with the approximation by a power law function. The power function used to approximate k_c_(h) has the general form

(9) $$k_{c}=k_{c1.1}h^{{-m}_{c}}$$

where k*_c_*_1.1_ is the cutting force needed to remove the cutting layer with a thickness of h = 1 mm and a width of b = 1 mm (unit cutting force), and m*_c_* is the exponent determined experimentally using the least squares method based on the results of the force measurements as a function of the thickness of the cutting layer.

Substituting (9) into (7) gives an engineering formula for the cutting force, the so-called Kienzle equation [42]:

(10) $$F_{c}=k_{c1.1}bh^{1{-m}_{c}}=k_{c1.1}bh^{y_{c}}$$

Equation (10) is used by engineers when determining, for example, the cutting power for selecting a machine tool, based on the constants from available catalogues or online calculators offered by tool manufacturers. It should be noted that Equation (10) can be used regardless of the type of machining process and blade geometry, which makes this equation universal.

**Figure 9:**
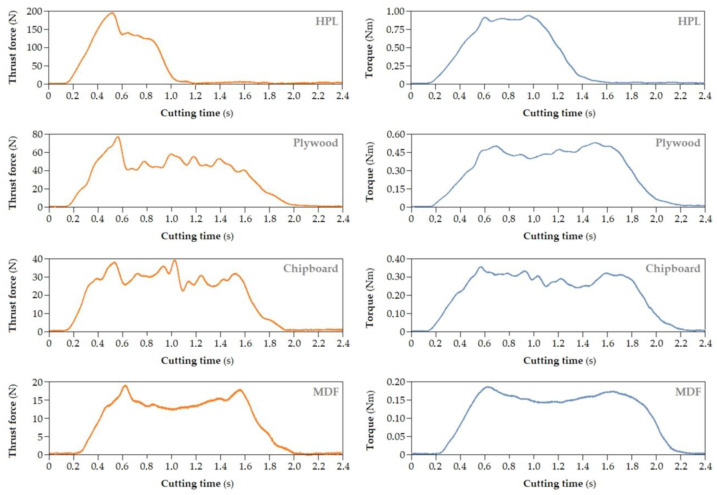
Waveforms of signals of the thrust force (**left**) and cutting torque (**right**) for the four processed materials; drilling process parameters: *n* = 3500 rpm and v_f_ = 750 mm/min.

Analyzing the course of the thrust force Ft and cutting torque M_s_ recorded during the tests (Figure 9), it can be noticed that the signals contain characteristic fragments responsible for the entry and exit of the tool from the material, at which the signals increase or decrease accordingly.

Force fluctuations related to the passage of the cutting tool through layers of the workpiece material of different densities can be distinguished in the signal. It was noticed that, during drilling, characteristic fragments of the thrust force F_t_ signal could be extracted. The first fragment is the area where the chisel edge of a grill enters the workpiece material; thus, the thrust force increases. Then, the drill starts cutting the outer layer of the material with the highest density. For HPL, this does not occur because the density of the material is similar throughout the entire cross-section of the board. When the drill leaves the area of the material with increased density, the force decreases because the drill cuts into the middle layer of lower density. Then, there is a signal fragment in which the drill penetrates to a depth equal to the height of the drill’s cutting blades. This is cutting with a constant cross-section of the cutting layer in the middle layer of the workpiece. This results in stabilization of the thrust force value. At the beginning of the next fragment of signal, the value of the thrust force begins to increase due to the fact that the drill begins to penetrate the outer layer of the material with a higher density.

When pressing the drill into the workpiece, the value of the cutting torque signal is approximately equal to 0. After the cutting process begins, the cutting torque value gradually increases until the maximum cross-section of the cutting layer is reached. After this stage, the torque signal stabilizes during cutting with a constant cross-section of the cutting layer. Finally, as the drill approaches the outer layers of materials with increased density, the cutting torque increases slightly.

When determining the cutting resistance, we must take into account the cutting torque M_s_ values during the drilling process (Figure 9). An application in the LabVIEW program was developed that allows the cutting torque value to be automatically determined from specific signal locations.

**Figure 10:**
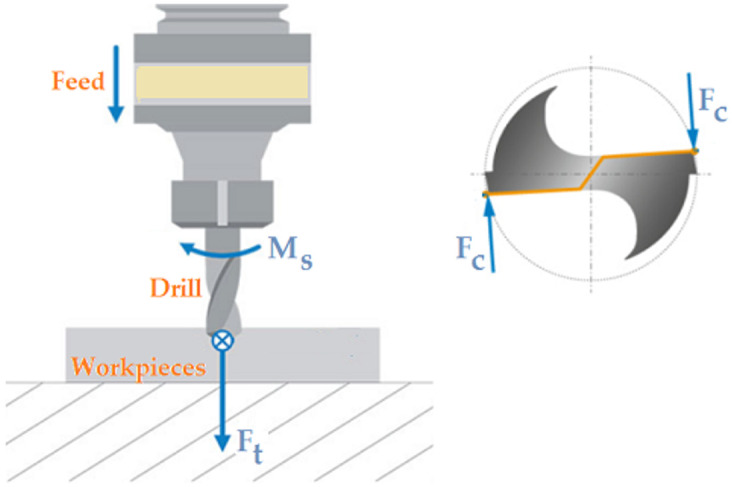
Distribution of the forces in the drilling process.

Using the cutting parameters shown in Table 3, drilling tests were performed on four workpiece materials. Figure 10 shows the distribution of forces occurring in the drilling process adopted in the research. In our case, there was only one force vector F_c_ resulting from the drill geometry shown in Figure 6a (one cutting edge).

As mentioned earlier, the specific cutting resistance is defined as the ratio of the cutting force to the cross-sectional area of the cutting layer (Equation (7)). To determine the cutting resistance k_c_ for the tested materials, it was necessary to determine the instantaneous value of the cutting torque M_s_ occurring in the drilling process. The cross-sectional area of the cutting layer was variable, depending on the feed speed values used. The cutting width b remained constant, while the thickness of the cutting layer changed depending on the drilling parameters; therefore, before determining the specific resistance, it was necessary to calculate the cross-sectional area of the cutting layer for all drilling tests using the equation

(11) $$A_{d}=h\cdots b$$

**Figure 11:**
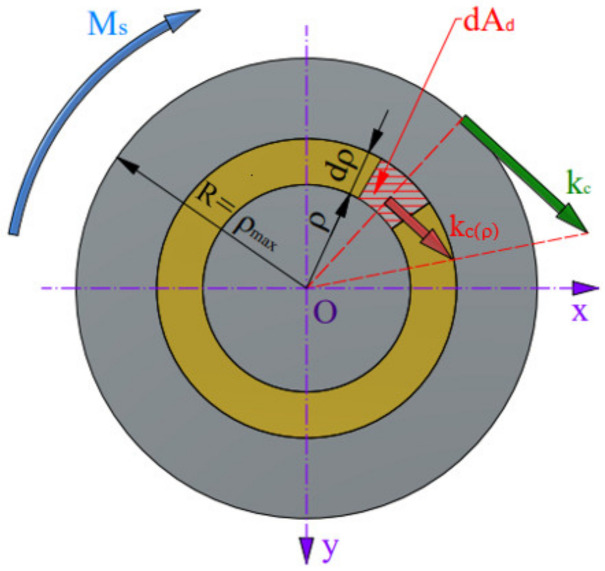
Distribution of the cutting resistance along the cutting edge of the drill.

Knowing the values of the cutting force F_c_ and the A_d_, the values of specific cutting resistance were determined at given drilling parameters for all the tested materials. For this purpose, the distribution of the cutting resistance values depending on the considered point on the cutting edge was described (Figure 11). 

As can be seen from Figure 11, the cutting resistance k_c_(ρ) acting on the infinitesimal element dA_d_ of the cross-section of the drill cutting edge gives the elementary torque dM_s_ relative to the drill axis:

(12) $${dM}_{s}=k_{c(\rho )}\cdot \rho \cdot {dA}_{d}$$

where k_c_(ρ) is the cutting resistance at considered radius ρ, and dA_d_ is the elementary area of the cutting layer. 

The equilibrium conditions show that the sum of these elementary moments collected over the entire length of the cutting edge of the drill must be equal to the moment M_s_ acting on the drill:

(13) $$M_{s}=\int_{A_{d}}{dM}_{s}=\int_{A_{d}}k_{c(\rho )}\cdot \rho \cdot {dA}_{d}$$

Taking into account the distribution of the cutting resistance along the cutting edge of the drill,

(14) $$\frac{k_{c(\rho )}}{k_{c}}=\frac{\rho }{\rho_{max}}$$

We determine k_c_(ρ) and, after substituting into Equation (13), we get

(15) $$M_{s}=\int_{A_{d}}k_{c}\cdot \frac{\rho }{\rho_{max}}\cdot \rho \cdot dA$$

The radius R as a constant for the entire integration area and the maximum cutting resistance k_c_ can be taken before the integral sign, and then,

(16) $$M_{s}=\frac{k_{c}}{\rho_{max}}\int_{A_{d}}\rho^{2}\cdot dA$$

The elementary surface area dA_d_ can be expressed as

(17) $${dA}_{d}=d\rho \cdot h$$

where h is the thickness of the cutting layer.

Substituting the Equation (17) into Equation (16), we get

(18) $$M_{s}=\frac{k_{c}\cdot h}{\rho_{max}}\int_{r}^{R}\rho^{2}\cdot d\rho$$

In our case, the radius $\rho_{max}$ is equal to the radius of the drill, and then,

(19) $$M_{s}=\frac{k_{c}\cdot h}{R}\int_{r}^{R}\rho^{2}\cdot d\rho$$

Integrating Equation (19) we get

(20) $$M_{s}=\frac{k_{c}\cdot h}{3\cdot R}\left(R^{3}-r^{3}\right)$$

After transforming Equation (20), we obtain the relationship determining the cutting resistance:

(21) $$k_{c}=\frac{3\cdot M_{s}\cdot R}{h\cdot (R^{3}-r^{3})}\;(N/{mm}^{2})$$

The cutting torque values, on the basis of which the specific cutting resistance was determined, are presented in Table 4. The cutting torque increased with the increase in the thickness of the cutting layer.

**Figure 12:**
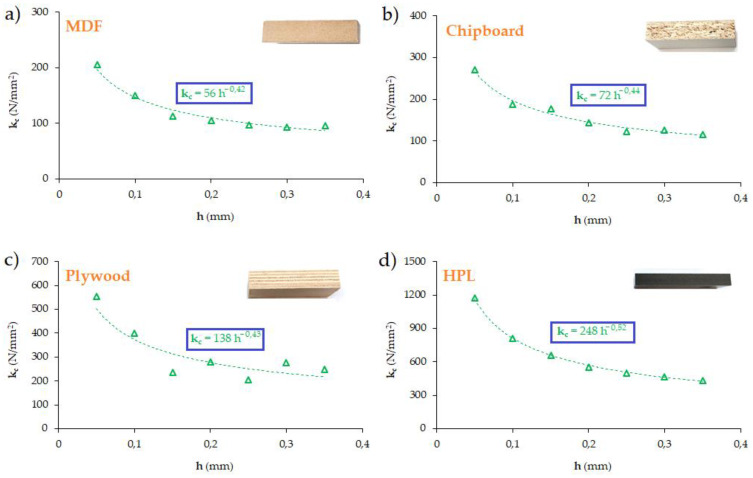
The unit cutting resistance in the drilling process: (**a**) MDF, (**b**) chipboard, (**c**) plywood board and (**d**) HPL.

The procedure for determining the cutting resistance from Equation (21) was repeated for all the tested materials. In this case, it was possible to obtain the value of the unit cutting resistance k_c1.1_ and the exponent m_c_ for each tested material. The charts and the parameters existing in Equation (9) are presented in Figure 12.

Table 5 presents the values of the unit cutting resistance determined in the conducted tests. As can be observed, the value of the specific cutting resistance is strongly dependent on the type of material.

Knowing the value of the unit cutting resistance allows the determination of the value of the force F_c_ based on the Kienzle equation (Equation (10)).

### 3.2. Dynamic Characteristics of the System

**Figure 13:**
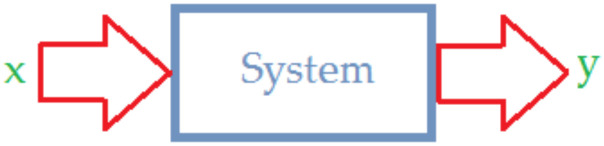
Data flow in a dynamic system.

An important stage in the analysis of the acceleration (Acc.) signal in the frequency domain is to determine the frequency characteristics of the analyzed system. When the system is excited by excitation x, we obtain the response y of the system to the existing excitation (Figure 13).

**Figure 14:**
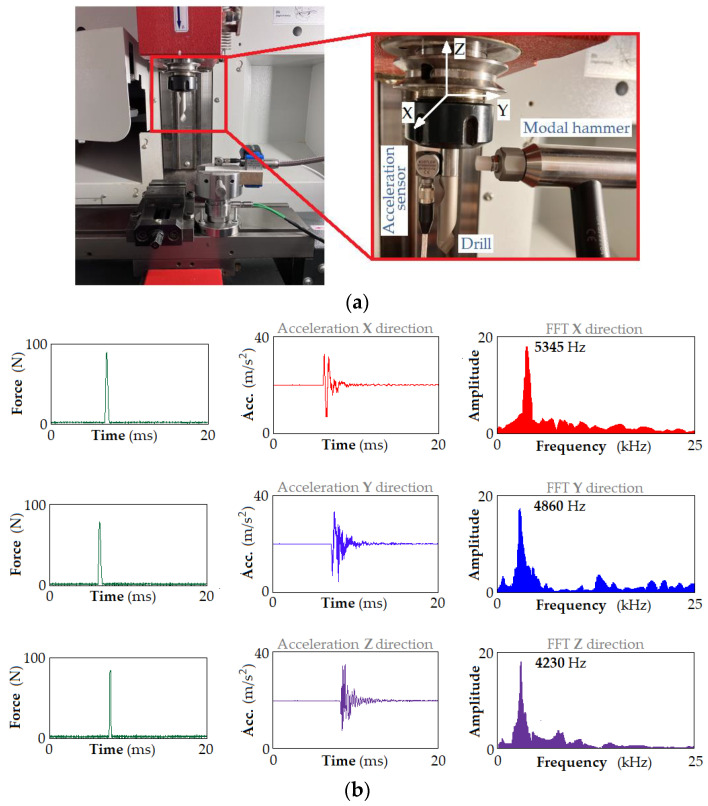
Dynamic analysis of the MHC system: (**a**) excitation of the MHC system using modal hammer and (**b**) the main natural frequencies of the MHC system.

The Dirac pulse (called the Dirac delta function) was used to determine the impulse response of the system (Figure 14). The Dirac delta function is approximated by a rectangular pulse, but it can have any shape, provided that the unit value of the integral $\int_{-\infty }^{+\infty }\delta \left(t\right)dt$ is maintained at 1 with the duration approaching zero.

The Fourier transform is a basic tool for spectral analysis, which entails searching for components with different frequencies in a signal. The Fourier transform can be interpreted as the correlation of the analyzed signal x(t) with the complex function e^−j2πft^, containing harmonic signals (cosine and sine) with frequency f: e^−j2πft^ = cos(2πft) – jsin(2πft).

A dynamic analysis of the machine-holder-cutting tool (MHC) system was carried out. For this purpose, the system was excited by an excitation in the form of a Dirac pulse (modal hammer) (Figure 14a). Knowing the forcing force signal and the system’s response in the form of acceleration, a modal analysis was performed to determine the natural frequency of the system (Figure 14). The onset of a single hit was then determined. This is recognized in the strength signal as exceeding a threshold that is five times the maximum value of the first 100 signal samples. Then, 750 samples are extracted [43]. Figure 14b shows an example of time waveforms and their spectra extracted from the entire signal.

### 3.3. Analysis of Short-Time Fourier Transform Signals

The acceleration signal was measured in both the transverse X, Y and axial Z directions. Acceleration signals on the analyzed directions contain information regarding the following:

- The vibrating length of the drill in the transverse and axial directions does not change during the drilling process, thus maintaining a rather constant frequency,
- Natural frequencies in the lateral and axial directions of the MHC system during drilling are essentially insensitive to the drill diameter, which simplifies monitoring for a wide range of drill sizes,
- The vibration in the X, Y and Z directions is influenced by the cutting torque and thrust force, which are the main sources of excitation during drilling.

#### 3.3.1. Signal Analysis in the Time Domain

**Figure 15:**
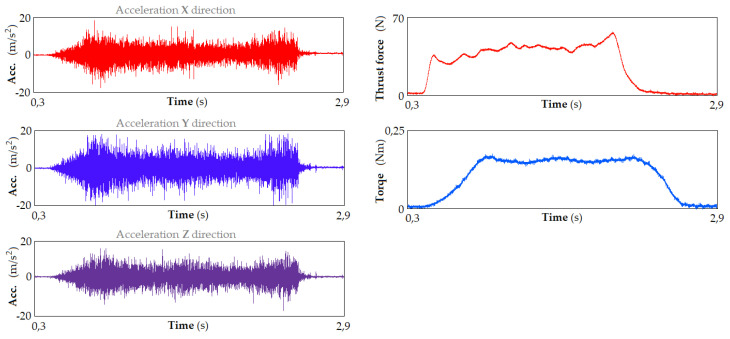
Acceleration signals (**left**) and signals of the thrust force and torque (**right**) in the time domain, registered for cutting speed 94 m/min and feed rate 700 mm/min.

Figure 15 shows the thrust force and torque signals, and the accelerometer (Acc) signals in the time domain in the X and Y (lateral) and Z (vertical) directions, recorded for the selected sample.

Drilling was carried out with the spindle speed of *n* = 3000 rpm, the feed rate of v_f_ = 750 mm/min and the drill diameter of 10 mm. As can be seen, the vibration signals can be characterized as consisting of short high–low frequency oscillatory transients occurring randomly over the duration of drilling. As the density of the cutting material increases, the amplitude of these signals increases. The excitation frequency of the MHC system in the drilling process is independent of cutting conditions such as feed rate and cutting speed. The majority of vibration signals consist of frequency components related to the dynamics of the cutting system.

**Figure 16:**
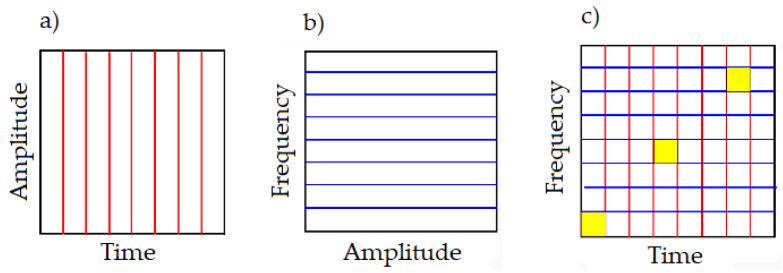
Comparison of analysis methods: (**a**) time (observation of signal properties in the time domain), (**b**) frequency (observation of signal properties in the frequency domain), and (**c**) STFT (observation of signal properties in the time–frequency plane).

Figure 16 illustrates the idea of three known signal analysis methods: time, frequency and time–frequency (short-time Fourier transform—STFT). It is clearly visible that, in the STFT method, the time–frequency resolution is fixed over the entire time/frequency plane (Figure 16c). When analyzing the signal using the fast Fourier transform (FFT), we do not have any information about changes in the frequency of the signal being examined over time (Figure 16b). In some cases, this information is very helpful. 

STFT allows the recovery of the time information that is lost when using FFT. STFT is characterized by a relatively short computation time. By selecting the appropriate sampling time and the length of the time window, it is possible to optimize the method to obtain the greatest ‘sensitivity’ in the frequency area on which the analysis is focused. The main disadvantage of the method is the fixed size of the time window. This results in the quality of time information being inversely proportional to the quality of the frequency information, which means that a higher resolution of one parameter worsens the accuracy of the other.

**Figure 17:**
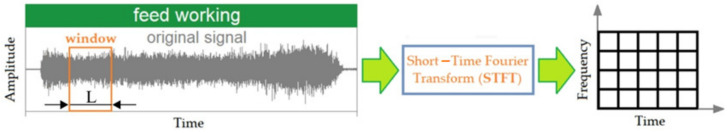
A floating time window.

STFT enables the extraction of information from a signal about how its spectrum changes over time, that is, the simultaneous observation of signal properties in both the time and frequency domains. The signal window (samples in the range L) intended for analysis is divided into segments, and each segment is independently analyzed spectrally. Successive ‘shifting of the time window’ allows the localization of the spectral parameters of the signal in time (Figure 17).

The use of a floating (movable) time window allows the determination of the phase content of a signal as it changes over time. Mathematically, STFT can be written as

(22) $$STFT\left\{x(t)\right\}=X\left(\tau ,\omega \right)=\int_{-\infty }^{\infty }x\left(t\right)\omega (t-\tau )e^{-j\omega \tau }dt$$

where x(t) is the analyzed signal and ω(t) describes the time window function. 

By moving the window in time along the signal, its spectral content is determined within a time interval whose length is determined by the window width. The shape of the time window plays a key role in STFT. The product of the window width (L) in the time domain and the window width in the frequency domain is a constant value for a given window. Hence, by improving the resolution in the time domain, it will deteriorate in the frequency domain and vice versa. Therefore, the window width is chosen as a compromise. STFT provides information about whether and when a given frequency component is in the time domain. However, this information has limited precision depending on the size of the time window in which the analysis is performed.

The square of the STFT amplitude a”lows’us to obtain the spectrogram function:

(23) $$spectrogram\left\{x(t)\right\}={\left|X\left(\tau ,\omega \right)\right|}^{2}$$

To analyze the recorded acceleration signals, a custom application was created in the LabVIEW program to enable the determination of selected values of the recorded signals at selected time intervals. The application worked by automatically determining the values of recorded signals at precisely defined time intervals.

It is obvious that most of the recorded acceleration signals do not show clearly characteristic features related to the transition from drilling in the MDF board to drilling in the HPL. However, after analyzing the acceleration signals in the frequency domain, a characteristic frequency range could be observed, where an increased value of the amplitude signal was the dominant feature depending on the material being processed. To obtain frequency ranges characteristic for the type of material being processed, short time intervals were analyzed and the signal spectra were determined. ‘Windowing’ involves the convolution of the input signal and the time window function in the time domain. The result of the above operation is a change in the signal amplitude as a window function. The time window is a function that describes how samples are obtained from the analyzed signal. Assuming that a certain signal s(n) is given in a finite time interval, then the result of observing such an impulse in the time window will be the function g(n) described by the following formula:

(24) $$g\left(n\right)=s\left(n\right)w\left(n\right),\;n\in (-\infty ;+\infty )$$

where w(n) is the mentioned window function.

A special example of a time window is the Hamming window. Developed by R. W. Hamming, it minimizes the maximum value of the nearest side lobes and is characterized by the following formula:

(25) $$w\left(n\right)=\alpha -\beta cos(\frac{2\pi n}{N-1})$$

where α = 0.54, β = 1 – α = 0.46 and *N* is the number of signal samples.

Obtaining a spectrogram enabling effective signal identification requires the appropriate selection of parameters such as window width, window function and time domain resolution. The highest resolution in the time domain can be obtained by using an overlap of N – 1 samples, but moving the window in each step by only one sample is associated with a significant increase in the time required for the calculations. Therefore, in the conducted research, an overlap of 50% of the window length was used. The proper selection of the window length is a slightly more complex issue. The highest efficiency was achieved when the ratio of the mean square frequency of length A to the duration B was equal to the ratio of the frequency increase to the time during which the increase took place:

(26) $$\frac{A}{B}=\frac{∆f}{∆t}$$

The problem of selecting the type of window function and its parameters should be a compromise between the signal quality obtained at the output and the time necessary to perform the calculations. Two basic parameters are used to evaluate the properties of the windows used in the STFT transform: the main lobe width and maximum height of side lobes of their spectrum determined using the basic Fourier transform. It should also be noted that the selection of the window function itself is a compromise between the width of the main lobe, the level of the first side lobe and the speed of change of side lobe height with increasing frequency. Therefore, it is a compromise between the accuracy of the amplitude value and the frequency.

#### 3.3.2. Vibration Spectra in the Transverse X and Y Directions

**Figure 18:**
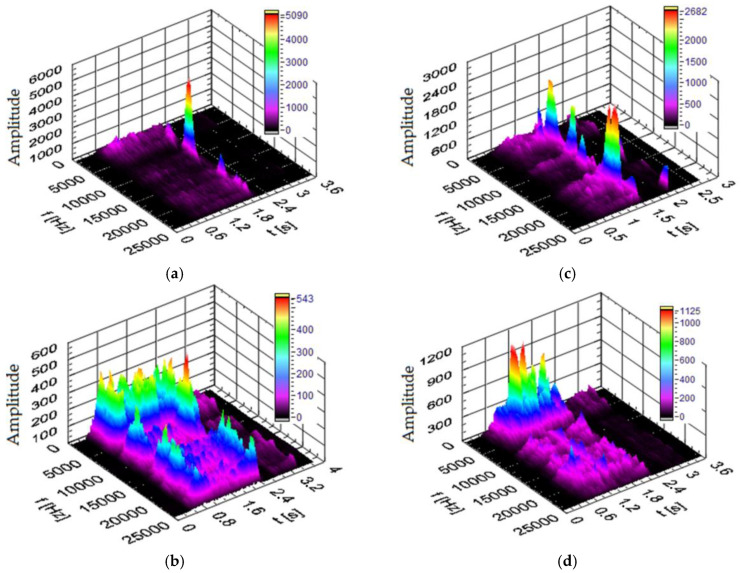
Spectrogram of the vibration signal in the X direction for (t—time, f—frequency) (**a**) plywood board, (**b**) HPL, (**c**) MDF and (**d**) chipboard.

**Figure 19:**
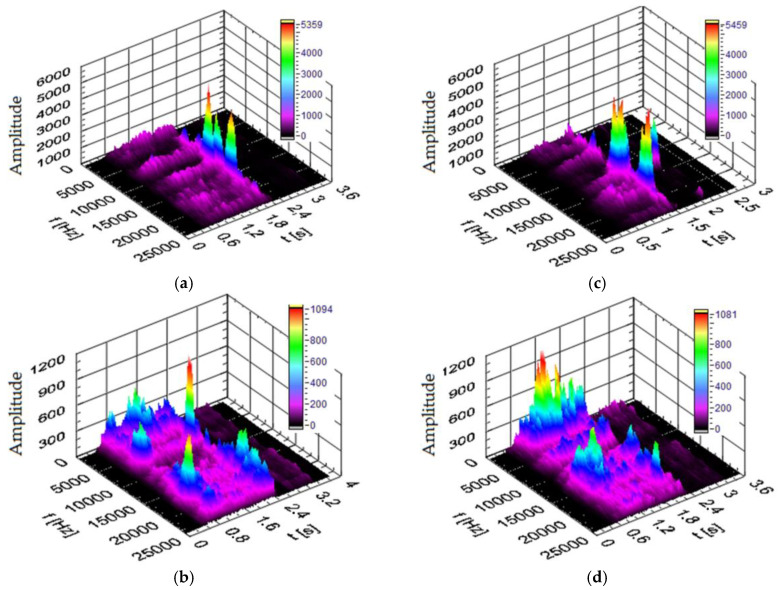
Spectrogram of the vibration signal in the Y direction for (t—time, f—frequency) (**a**) plywood board, (**b**) HPL, (**c**) MDF and (**d**) chipboard.

Figure 18 and Figure 19 show the power spectra of vibration signals generated in the X and Y directions during drilling in various types of workpiece materials. The cutting parameters were *n* = 3000 rpm and v_f_ = 750 mm/min. As shown in the figures, the maximum amplitude of the vibration signal in the frequency range varies in the range from 2 to 22 kHz, depending on the type of material being processed. It can be seen that, when drilling in plywood board (Figure 18a and Figure 19a), the power spectrum has characteristic increases in amplitude at frequencies of approximately 10 kHz in the X direction and 11 kHz and 12.5 kHz in the Y direction. When drilling in HPL (Figure 18b and Figure 19b), the power spectrum has characteristic increases in amplitude at frequencies of approximately 19 kHz in the X direction, and 19 kHz in the Y direction. When drilling in the MDF material (Figure 18c and Figure 19c), the power spectrum has characteristic increases in amplitude at frequencies of approximately 10 kHz in the X direction and 7.5 kHz in the Y direction. When drilling in a chipboard (Figure 18d and Figure 19d), the power spectrum has characteristic increases in amplitude at frequencies of approximately 2.5 kHz in the X direction and 2 kHz in the Y direction. Comparing the obtained values of the amplitude of the dominant frequency, depending on the material being processed, it can be concluded that there is a significant impact of the processed material on the range of the dominant frequency of the signal. It can also be observed that there is a correlation between the hardness of the workpiece material and the dominant frequency range in the vibration signal. For example, for the HPL, the value of the dominant frequency is approximately 19 kHz (material with the highest hardness) and, for chipboard, it is approximately 2.5 kHz (material with the lowest hardness).

Figure 14b shows the main natural frequencies of the MHC system obtained during dynamic analysis of this system. It is obvious that the high-frequency components in the vibration signals observed in the signals do not correspond to one or more of the natural frequencies of the machine spindle and the frequencies of the workpiece mounting system [43]. The above analysis did not consider the effect of cutting edge wear on the vibration signal signature.

#### 3.3.3. Vibration Spectra in the Z Direction

**Figure 20:**
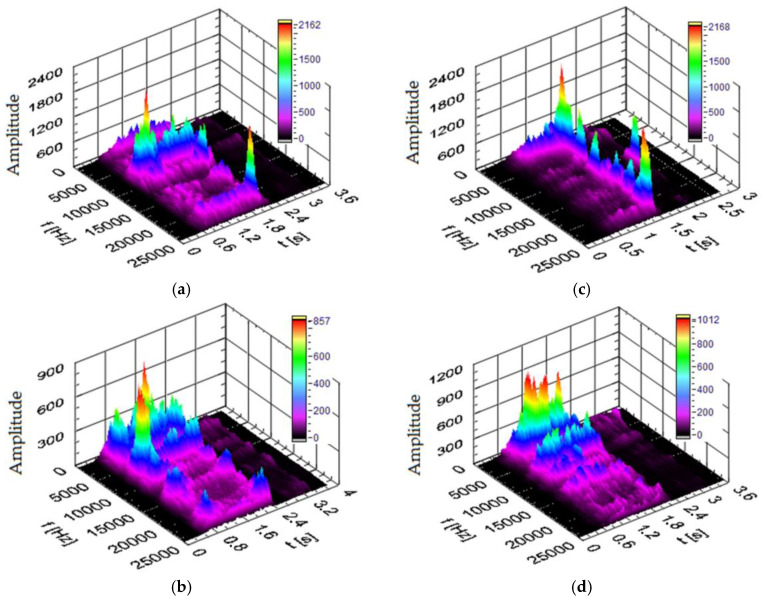
Spectrogram of the vibration signal in the Z direction for (t—time, f—frequency) (**a**) plywood board, (**b**) HPL, (**c**) MDF and (**d**) chipboard.

Figure 20 shows the power spectra of the vibration signal in the Z direction obtained for the different materials processed in the drilling process. The cutting conditions were the same as those used in the tests in the X and Y directions. The vibration spectra resulting from the type of material being machined in the Z direction are different from those obtained in the X and Y directions. As shown in Figure 20, different areas of frequency ranges were excited during drilling. Two characteristic amplitude peaks are visible in the analyzed signals. When drilling in plywood board (Figure 20a), the signal of power spectrum has characteristic increases in amplitude at frequencies around 8 kHz and 22 kHz. When drilling in HPL (Figure 20b), the power spectrum has characteristic increases in amplitude at frequencies around 4 kHz and 22 kHz. When drilling in MDF material (Figure 20c), the power spectrum has characteristic increases in amplitude at frequencies around 3 kHz and 8 kHz. When drilling in a chipboard (Figure 20d), the power spectrum has a characteristic increase in amplitude at frequencies of approximately 2.5 kHz in the Z direction. 

By comparing the values of the amplitude of the dominant frequency, a significant influence of the processed material on the dominant frequency range in the signal can be distinguished. It is not as clear as in the case of the signal spectra in the X and Y directions (Figure 18 and Figure 19). However, a significant correlation between the hardness of the processed material and the dominant frequency range in the vibration signal can no longer be observed.

In general, the vibration signals measured in the X, Y and Z directions are influenced by the type of material being processed. The results clearly show that vibration spectra can be used to identify the workpiece material. Various types of information can be extracted from the analysis of vibration signals in both the time and frequency domains. These results show that there are specific differences related to the material removal mechanism between drilling processes of different materials, detectable by acceleration signals. This means that evaluating the signal in the frequency domain will enable clear identification of the material.

### 3.4. Permutation Entropy

Measuring the time series complexity of a dynamic system is an interesting topic in science and engineering because its complexity is related to complex internal patterns hidden in the system’s dynamics. If there is no recognizable structure in a system, it is considered stochastic. Permutation entropy (PermEn) refers to the local order of the structure of a time series that gives quantitative measures of complexity. The mathematical details of the PermEn method can be found in [6,44]. The concept of PermEn was introduced by Bandt and Pompe [44] to arrange subsequent values of a time series, neglecting the scale of the differences between them. Moreover, Keller et al. [45] described the permutation analysis approach.

The permutation entropy of a time series is the Shannon entropy of the distribution of order patterns in the time series [46]. Such ordinal patterns, describing the ordinal types of vectors, are encoded by Permutations. Let us denote the set of permutations {1, …, d} for d belonging to the natural numbers by Π_d_. We say that the vector X defined by the elements x(1), x(2), …, x(d) has the ordinal formula π = (π_1_, …, π_d_) ∈ Π_d_, which orders these elements:

x(π_1_) < x(π_2_) < … < x(π_d_) (27)

If x(i) = x(j), we determine that x(i) < x(j), when i < j. The elements of the set Π_d_ are divided into overlapping vectors of length d according to the following scheme:

(x(1), …, x(d)), ((x(2), …, x(d+1)), …, (x(N − d+1), …, x(N))
(28)

A specific pattern is assigned to each vector according to Equation (27), thanks to which we obtained a distribution of ordinal patterns. The number of possible patterns of length d is d!. The main application of pattern decomposition is to calculate entropy as a measure of the disorder of a system, that is, PermEn. The distribution of PermEn is determined empirically by counting the probability of each pattern occurring in the entire sequence N of data according to the formula

(29) $$p\left(\pi \right)=\frac{\left|\left\{n:0\leq n\leq N-d+1,\left(x\left(n\right),x\left(n+1\right),\ldots ,x\left(n+d-1\right)\right)is\;type\;\pi \right\}\right|}{N-d+1}$$

The PermEn formula is based on the Shannon entropy formula, but instead of the probability distribution of events, the following pattern of probability distribution was used:

(30) $$PermEn=-\sum_{i=1}^{d!}p\left(\pi_{i}\right)*ln\;p(\pi_{i})$$

The normalized PermEn (H_p_) is, therefore [47],

(31) $$H_{p}=\frac{H_{p}(d)}{ln(d!)}$$

The highest possible value of H_p_ is 1, which means that all permutations have equal probability. The smallest possible value of H_p_ is 0, which means that the time series is very regular. In short, the smaller the value of H_p_, the more regular the time series. The choice of n and d depends on the time series. Equation (31) indicates that the obtained probability distribution can be characterized by parameter d (dimension). It plays an important role in assessing the appropriate probability distribution because d determines the number of available states given by d!. Furthermore, to obtain reliable statistics and a proper distinction between stochastic and deterministic dynamics, it is necessary that N ≫ d! [48]. For practical reasons, Bandt and Pompe [44] suggested choosing the parameter d in the range 3 ≤ d ≤ 7 d. Permutation entropy can be used to distinguish chaotic data from noise in timescale identification [49].

The PermEn for the thrust force signal for the processed materials was calculated in a window of 1000 elements, moving in increments of 200. Changing the signal frequency affects the PermEn value. It increases linearly with the increase in the signal frequency. PermEn is very sensitive to adding noise of different strengths. To eliminate the effects of unexpected noise in the signal, filters were used to process the signal’s features before applying it to detect the change in the workpiece material in the drilling process.

**Figure 21:**
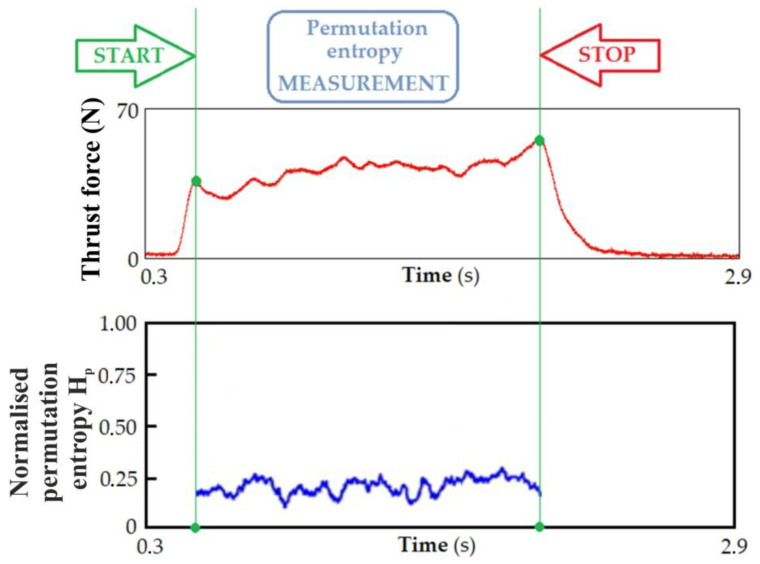
Variation of the thrust force signal and PermEn H_p_.

Figure 21 shows the thrust force signal and its PermEn H_p_. The PermEn H_p_ values are low for materials with a homogeneous structure under normal cutting conditions because the force signal is similar to a regular periodic signal. However, the thrust force signals have different waveforms due to various effects, including different material density, hardness, and coefficients of friction in different positions. The H_p_ values changed within relatively small limits in the drilling processes of the materials tested. Table 6 shows the change in H_p_ values for the processed materials. The results show that the PermEn of thrust force signals can be applicable and indicate a material change in the drilling process.

## 4. Conclusions

This paper presented the results of the investigations on the possibility of material identification in the drilling process of wood-based materials based on selected measures of the thrust force and the cutting torque signals, as well as the vibroacoustic signal. Based on the research results obtained, the following can be concluded:

- Identification of the material being processed during the drilling process is possible based on both cutting force F_c_, cutting torque M_c_ and acceleration signals.
- Identification based on the cutting force and cutting torque signals is based on the value of the unit cutting resistance k_c1.1_ and on the basis of the change in the value of PermEn H_p_.
- Identification based on STFT analysis of the acceleration signals in specific directions X, Y and Z uses the assessment of the dominant frequency amplitudes depending on the material being processed. There is no need to know the signal history to be able to identify the processed material.
- This article presents the usefulness of the cutting torque signal in the drilling process of wood-based materials, with the aim of identifying the material during the cutting process. As expected, the unit cutting resistance k_c1.1_ varied in value depending on the type of material being processed, which allowed for a clear distinction between materials. The results show that the proposed methodology can be used as an intelligent technique in the drilling process to identify machined materials.
- Additionally, the applied material identification based on changes in the PermEn H_p_ value of the cutting force F_c_ signal during the drilling process worked reliably for all processed materials analyzed. This measure turned out to be insensitive to the combinations of drilling parameters used in the investigations. The proposed method enables the reliable detection of tool contact with the workpiece material and identification of the material during the drilling process.
- There are no clearly visible differences in the recorded vibroacoustic signals in the time domain when changing the processed material. However, after transforming the signal into the frequency domain, characteristic frequency ranges with dominant amplitude can be observed depending on the material being processed.
- A methodology is proposed that can be used as an intelligent technique to support the drilling process in order to detect the material being processed using data from sensors installed on the machine tool. Typically, in the woodworking industry, processing parameters are selected to correspond to the most difficult to process material in stacks at runtime, which increases cutting time, efficiency and processing quality. Intelligent machining techniques contribute to the real-time adaption of cutting parameters to the identified material.

## Figures and Tables

**Table 1:** Selected mechanical and physical properties of the test materials (values in parentheses are standard deviations).

Test Material	Density (kg/m^3^)	Bending Strength (MPa)	Elasticity Modulus (MPa)	Janka Hardness (N)
Plywood board	650 (32)	85 (9)	5600 (261)	3228 (189)
MDF	730 (23)	38 (4)	2530 (176)	4932 (245)
HPL	1470 (29)	110 (13)	9250 (349)	-
Chipboard	690 (19)	12 (3)	1850 (138)	2367 (153)

**Table 2:** Machining parameters of the drilling process.

Cutting Speed v_c_ (m/min)	Feed Per Tooth f_z_ (mm)	Feed Rate v_f_ (mm/min)	Rotational Speed of Tool n (rpm)
78	0.15	375	2500
	0.20	450	
	0.25	525	
94	0.15	500	3000
	0.20	600	
	0.25	700	
109	0.15	625	3500
	0.20	750	
	0.25	875	

**Table 3:** Machining conditions during redrilling.

Thickness of the Cutting Layer b (mm)	Feed Per Tooth f_z_ (mm)	Feed Rate v_f_ (mm/min)
0.05	0.07	177
0.10	0.14	354
0.15	0.21	530
0.20	0.28	707
0.25	0.35	884
0.30	0.42	1061
0.35	0.49	1237

**Table 4:** Cutting torque values.

**Test Material**	**Thickness of the Cutting Layer (mm)**						
	0.05	0.10	0.15	0.20	0.25	0.30	0.35
	**Cutting torque (Nm)**						
Plywood board	0.21	0.32	0.28	0.40	0.47	0.66	0.71
MDF	0.08	0.12	0.15	0.17	0.20	0.24	0.28
HPL	0.45	0.62	0.80	0.87	0.99	1.08	1.18
Chipboard	0.11	0.15	0.22	0.23	0.26	0.31	0.33

**Table 5:** The value of the unit cutting resistance k_c1.1_ and the exponent m_c_.

Workpiece Material	k_c1.1_ (N/mm^2^)	m_c_
MDF	56	−0.42
Chipboard	72	−0.44
Plywood board	138	−0.43
HPL	248	−0.52

**Table 6:** Normalized PermEn H_p_.

Test Material	H_p_
MDF	0.32
Chipboard	0.53
Plywood board	0.48
HPL	0.21

## Data Availability

Data are contained within the article.
